# Osimertinib and palbociclib in an *EGFR*-mutated NSCLC with primary *CDK4* amplification after progression under osimertinib

**DOI:** 10.1038/s41698-024-00607-9

**Published:** 2024-05-22

**Authors:** Vincent D. de Jager, Jos A. Stigt, Maarten Niemantsverdriet, Arja ter Elst, Anthonie J. van der Wekken

**Affiliations:** 1 Department of Pathology and Medical Biology, University of Groningen, University Medical Center Groningen, Groningen, The Netherlands; 2 Department of Respiratory Medicine, Isala Hospital, Zwolle, The Netherlands; 3 Department of Pathology, Isala Hospital, Zwolle, The Netherlands; 4 Department of Pulmonary Diseases and Tuberculosis, University of Groningen, University Medical Center Groningen, Groningen, The Netherlands

**Keywords:** Molecular medicine, Non-small-cell lung cancer

## Abstract

Precision cancer medicine has changed the treatment paradigm of patients with non-small cell lung cancer (NSCLC) with specific molecular aberrations. A major challenge is management of the resistance that tumor cells eventually develop against targeted therapies, either through primary or acquired resistance mechanisms. We report a 61 year-old male patient with metastatic NSCLC harboring an *EGFR* exon 19 deletion, a *PIK3CA* mutation, and *CDK4* amplification. After an initial partial response to osimertinib as mono-therapy (third-generation EGFR tyrosine kinase inhibitor), the patient had progression of disease after 4 months of treatment and was referred for combined osimertinib and palbociclib (CDK4/6 inhibitor) treatment. Though complicated by transient pneumonitis, the patient has an ongoing partial response for > 10 months and has experienced clinical improvement on this treatment regimen. As amplification of *CDK4* occurs in ~ 10% of treatment-naïve patients with EGFR-mutated NSCLC, the successful treatment of our patient with osimertinib and palbociclib may be highly relevant for future patients with NSCLC.

## Introduction

Mutations in epidermal growth factor receptor (*EGFR*) occur in 12–15% of European patients with non-small cell lung cancer (NSCLC) and 30–50% in Asian patients with NSCLC^1,2^. The current standard-of-care in patients with advanced stage NSCLC harboring a sensitizing *EGFR* mutation, excluding exon 20 insertion mutations, is first-line systemic treatment with a first-, second-, or third-generation EGFR tyrosine kinase inhibitor (TKI) with or without monoclonal antibodies (e.g., bevacizumab and ramucirumab), dependent on the specific *EGFR* mutation(s)^3^. For the two most common *EGFR* mutation groups (i.e., exon 19 deletion or L858R), the preferred first-line treatment is osimertinib, a third-generation EGFR TKI or erlotinib-ramucirumab, a combination of first-generation EGFR TKI and a VEGFR2 inhibitor^3^. Based on the FLAURA2 trial results, this standard-of-care for patients with common EGFR-mutated metastatic NSCLC may change to combined osimertinib and chemotherapy as first-line treatment^4^.

As with other targeted therapies, patients with metastatic EGFR-positive NSCLC treated with osimertinib will inevitably experience progression of their disease. Disease progression may be caused by primary resistance, in which co-occurring alterations bypass the effect of osimertinib or prevent binding to the mutated protein, or by acquired resistance, due to the development of additional molecular aberrations in tumor cells during exposure to a specific targeted therapy. Examples of common acquired resistance mechanisms to osimertinib include *EGFR* C797X mutations and amplification of mesenchymal-epithelial transition (*MET*)^5^. Examples of primary resistance mechanisms to osimertinib are phosphatidylinositol-4,5-biphosphate 3-kinase catalytic subunit alpha (*PIK3CA*) mutations, tumor protein 53 (*TP53*) mutations, but also aberrations of cell cycle genes, including amplification of cyclin-dependent kinase 4 (*CDK4*) or cyclin-dependent kinase 6 (*CDK6*)^6,7^. Pretreatment presence of *CDK4* or *CDK6* amplification has previously been associated with de novo resistance to first- or second-generation EGFR TKI in patients with sensitizing EGFR-mutated NSCLC^6^. Importantly, amplification of *CDK4* occurs in ~10% of treatment-naïve patients with EGFR-mutated metastatic non-small cell lung cancer^8,9^.

**Fig. 1:**
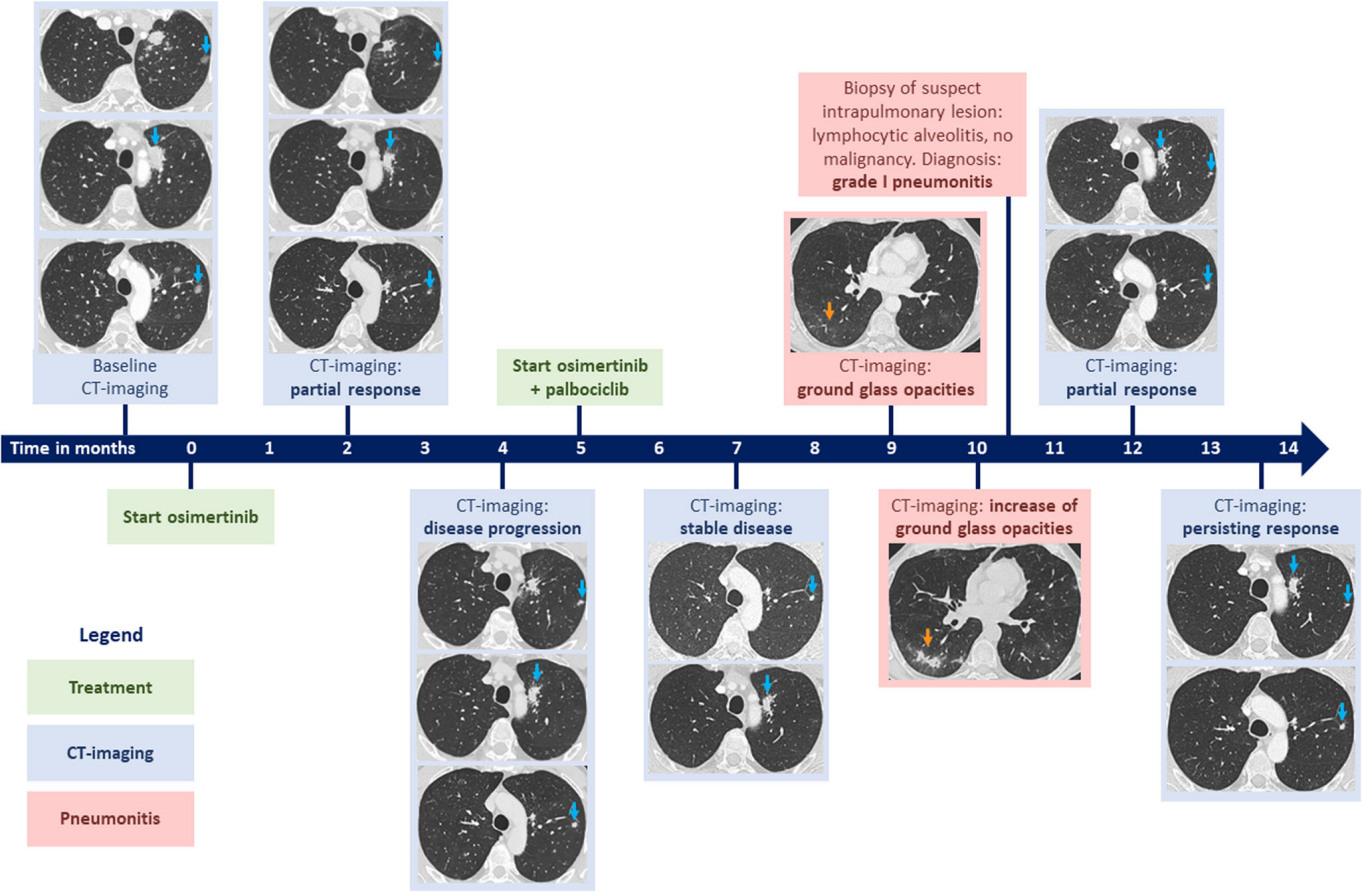
Timeline of treatment initiation and CT-imaging evaluation. The timeline represents the time of starting TKI treatment with osimertinib followed by adding palbociclib to osimertinib (green boxes). CT images have been provided at different timepoints for response evaluation (blue boxes). During treatment a pneumonitis occured (red boxes). The arrowhead represents ongoing response.

Here, we describe a patient with newly diagnosed metastatic NSCLC with a common *EGFR* mutation, a *PIK3CA* mutation and *CDK4* amplification, who benefited from combined osimertinib and palbociclib treatment after disease progression on treatment with osimertinib alone. The timeline of therapy initiation and treatment outcome is displayed in Fig. 1.

## Case presentation

A 61-year-old male patient with no prior oncological medical history was diagnosed with pulmonary adenocarcinoma of the upper lobe of the left lung with intrapulmonary and bone metastases (T4N2M1c).

Routine next-generation sequencing (NGS) was performed with Ion Torrent Oncomine focus DNA panel: hotspot primer sets for 35 genes (*AKT1*, *ALK*, *AR*, *BRAF*, *CDK4*, *CTNNB1*, *DDR2*, *EGFR*, *ERBB2*, *ERBB3*, *ERBB4*, *ESR1*, *FGFR2*, *FGFR3*, *GNA11*, *GNAQ*, *HRAS*, *IDH1*, *IDH2*, *JAK1*, *JAK2*, *JAK3*, *KIT*, *KRAS*, *MAP2K1*, *MAP2K2*, *MET*, *MTOR*, *NRAS*, *PDGFRA*, *PIK3CA*, *RAF1*, *RET*, *ROS1* and *SMO*) and copy number variant primer sets for 19 genes (*ALK*, *AR*, *BRAF*, *CCND1*, *CDK4*, *CDK6*, *EGFR*, *ERBB2*, *FGFR1*, *FGFR2*, *FGFR3*, *FGFR4*, *KIT*, *KRAS*, *MET*, *MYC*, *MYCN*, *PDGFRA* and *PIK3CA*). With estimated tumor cell percentage of 30%, molecular testing revealed the presence of an *EGFR* (ref. seq. NM_005228.5) c.2235_2249del15 (p.Glu746_Ala750del) mutation (classic exon 19 deletion mutation) with a variant allelic frequency (VAF) of 22%, a *PIK3CA* (ref. seq. NM_006218.3) c.3140 A > G (p.His1047Arg) mutation with a VAF of 17%, and an amplification of *CDK4* (ref. seq. NM_000075.3) of 21 copies. No sequential RNA analysis was performed due to the detected *EGFR* mutation. PD-L1 expression by immunohistochemical staining (22C3 antibody) was > 50%. Due to the presence of these co-occurring molecular aberrations in addition to the *EGFR* mutation, the patient was discussed in the regional Molecular Tumor Board of the University Medical Center Groningen (UMCG-MTB). Preceding the review of the patient in the MTB, first-line treatment with osimertinib at standard dose (80 mg per day) was started. The recommendation of the MTB was to await the response to the initiated treatment with osimertinib, and to refer the patient upon disease progression for combined treatment with osimertinib and palbociclib.

After 2 months of osimertinib treatment, the patient had a partial response on CT-imaging by RECIST criteria (v1.1)^10^. After 4 months of treatment, however, progressive disease was observed due to an increase in size and density of the intrapulmonary metastases. The primary tumor and bone metastases remained unchanged and no new lesions were observed. In response, the patient was referred to the UMCG for treatment with osimertinib and palbociclib. At the time of referral, the patient experienced pain in his lower back with radiation to his left leg. Based on CT-imaging, these symptoms were considered to be caused by a vertebral compression fracture due to bone metastases, and were treated conservatively. The patient did not experience any pulmonary symptoms.

Five months after initial start of osimertinib treatment, palbociclib was requested for compassionate use and was added to the treatment regimen in treatment bouts of 3 weeks followed by 1 week without palbociclib. Osimertinib was continued daily at standard dosage of 80 mg per day. Two months after the start of the combination therapy regimen, response to treatment was considered stable disease on CT-imaging. The primary tumor in the left upper lobe had slightly decreased in size and the intrapulmonary metastases in both lungs had either decreased in size or were unchanged compared to prior imaging. Bone metastases remained unchanged and no new lesions were observed. Medication-related toxicities included grade II leucopenia and neutropenia, and grade I thrombocytopenia and skin rash. Four months after start of combination therapy, CT-imaging revealed pulmonary abnormalities in both lungs consisting of ground glass opacities. The patient did not experience any pulmonary symptoms, such as dyspnea or coughing. Differential diagnosis consisted of pneumonitis, medication-induced or viral cause, or disease progression. In the absence of pulmonary symptoms, imaging was repeated after 4 weeks, revealing an increase in number and size of irregular consolidations and nodules in both lungs in combination with progressing ground glass opacities. The patient was discussed in a multidisciplinary pulmonary oncology meeting, concluding there was suspicion for progressive disease in addition to pneumonitis. The patient was scheduled for a transthoracic biopsy of a lesion in the lower lobe of the right lung, suspected for disease progression.

In the biopsy, lymphocytic alveolitis with limited involvement of lymphocytes, histocytes and eosinophilic granulocytes was observed by the pathologist. In the absence of malignant cells in the biopsy and no pulmonary symptoms, a diagnosis of grade I pneumonitis was made^11^. Combination treatment with osimertinib and palbociclib was continued with unchanged treatment schedule and dosage. In the following 3 months, CT-imaging revealed a reduction of pneumonitis-related airspace consolidations and ground-glass opacities, and further size reduction of target lesions with a partial response according to RECIST guidelines (v1.1)^10^. During his treatment, the patient experienced improvements of the pain in his lower back and left leg, thereby improving his functioning. He has been able to travel for vacations, including physically demanding activities, such as skiing. In accordance with the declaration of Helsinki, the patient provided (written) consent for the publication of this case report.

## Discussion and conclusions

Management of tumor resistance to osimertinib is a major challenge in the treatment of patients with EGFR-mutated NSCLC^12^. The co-occurrence of *CDK4* or *CDK6* amplification has previously been described as a biomarker of primary resistance to EGFR TKIs, including osimertinib^6,13^. CDK4 and CDK6 play an integral role in the cell cycle pathway by promoting cell division^14,15^. Under normal circumstances, these kinases are regulated through the expression of D-cyclins, including cyclin D1/2/3 (CCND1/2/3), which bind to CDK4/6 to form active cyclin D-CDK4/6 complexes^14,15^. In many cancer types, including lung cancer, molecular aberrations of one or more of these genes may occur, which contribute to cell proliferation and oncogenesis^14^. Specific CDK4/6-inhibitors, such as palbociclib and ribociclib, have been approved for the treatment of hormone receptor-positive metastatic breast cancer in combination with fulvestrant or an aromatase inhibitor^16^. However, in three single-agent CDK4/6 inhibitor pan-cancer trials with patient selection based on specific molecular aberrations of cell cycle genes, only limited clinical activity was observed with no partial or complete responses^17,18^.

One case report has described combined afatinib and palbociclib treatment, which was initiated in a patient with EGFR L861Q-mutated advanced stage squamous cell carcinoma of the lung due to the detection of *CDK4* amplification after prior systemic treatment with erlotinib, afatinib, pembrolizumab and nivolumab^19^. However, in this case report, the initial biopsy was not tested for the presence of *CDK4* amplification at diagnosis. Moreover, tumor re-biopsy was performed after immunotherapy, and followed by chemotherapy treatment combined with recombinant endostatin. Lastly, the reason for stopping erlotinib and afatinib treatment is not stated, and patient follow-up was limited to 2 months. As previously described in literature, some patients with (uncommon) EGFR-mutated NSCLC who have experienced disease progression on afatinib and are subsequently treated with chemotherapy, may respond to rechallenge with afatinib^20,21^. Therefore, it is uncertain whether the described response in the previous case report may have been attributable to re-sensitization of the tumor to afatinib treatment, rather than the addition of palbociclib to the therapy regimen.

To our knowledge, this is the first report of a patient treated with osimertinib and palbociclib, which was based on the co-occurrence of an *EGFR* exon 19 deletion and *CDK4* amplification. Our patient had a short-lived partial response to first-line mono-osimertinib treatment. With combined osimertinib and palbociclib treatment as second line of treatment, he has an ongoing partial response at 10 months of follow-up. Four months after the addition of palbociclib to osimertinib treatment, the patient experienced transient, non-symptomatic pneumonitis. Both for osimertinib and palbociclib, interstitial lung disease or pneumonitis has been reported as potential toxicity. The prevalence of pneumonitis in patients with advanced stage NSCLC who are treated with EGFR TKI has been described as 1.1%^22^. The prevalence of pneumonitis in patients who are treated with palbociclib is unknown in patients with advanced stage NSCLC. In patients with advanced stage breast cancer who are treated with a CDK4/6 inhibitor, the prevalence of interstitial lung disease or pneumonitis has been described as 1.6%^23^. Combined osimertinib and palbociclib treatment may carry an increased risk for medication-induced pneumonitis.

In conclusion, based on the findings described in this unique case report, combined osimertinib and palbociclib treatment may be an effective treatment option for patients with metastatic EGFR-mutated NSCLC with co-occurring amplification of *CDK4*. Given the short-lived initial response of our patient to osimertinib, it may be considered to initiate combined osimertinib and palbociclib treatment as first line of treatment rather than to await disease progression on osimertinib monotherapy. Additionally, combined osimertinib and palbociclib treatment may be considered if *CDK4* amplification occurs as an acquired resistance mechanism. Patients receiving this combination of targeted therapies should be carefully evaluated for potential medication-induced pneumonitis, which may occur more frequently than with either targeted therapy alone.

## Methods

### Consent to publish

In the Netherlands, medical ethical board approval is not required for the publication of a case report. In accordance with the declaration of Helsinki, the patient provided (written) consent for the publication of this case report.

### NGS and PD-L1 IHC

Routine NGS analysis was performed on baseline biopsy with Ion Torrent Oncomine focus DNA panel: hotspot primer sets for 35 genes (*AKT1*, *ALK*, *AR*, *BRAF*, *CDK4*, *CTNNB1*, *DDR2*, *EGFR*, *ERBB2*, *ERBB3*, *ERBB4*, *ESR1*, *FGFR2*, *FGFR3*, *GNA11*, *GNAQ*, *HRAS*, *IDH1*, *IDH2*, *JAK1*, *JAK2*, *JAK3*, *KIT*, *KRAS*, *MAP2K1*, *MAP2K2*, *MET* (no exon 14 skipping), *MTOR*, *NRAS*, *PDGFRA*, *PIK3CA*, *RAF1*, *RET*, *ROS1* and *SMO*) and copy number variant primer sets for 19 genes (*ALK*, *AR*, *BRAF*, *CCND1*, *CDK4*, *CDK6*, *EGFR*, *ERBB2*, *FGFR1*, *FGFR2*, *FGFR3*, *FGFR4*, *KIT*, *KRAS*, *MET*, *MYC*, *MYCN*, *PDGFRA* and *PIK3CA*). Routine immunohistochemical staining for PD-L1 was performed on baseline biopsy (antibody 22C3).

### Clinical data

Follow-up data were retrieved from the electronic patient file. Response evaluation was performed by CT-imaging and assessed according to RECIST guidelines (v1.1). Representative CT-images of tumor foci at follow-up timepoints were used for Fig. 1. Figure 1 was created with Microsoft PowerPoint (Version 2402).

### Reporting summary

Further information on research design is available in the Nature Research Reporting Summary linked to this article.

### Supplementary information

Reporting Summary

## Data Availability

Not applicable. All available data are stated in this case report.
